# The Association between Frailty Indicators and Blood-Based Biomarkers in Early-Old Community Dwellers of Thailand

**DOI:** 10.3390/ijerph16183457

**Published:** 2019-09-17

**Authors:** Warathit Semmarath, Mathuramat Seesen, Supachai Yodkeeree, Ratana Sapbamrer, Pisittawoot Ayood, Rungnapa Malasao, Penprapa Siviroj, Pornngarm Limtrakul (Dejkriengkraikul)

**Affiliations:** 1 Department of Biochemistry, Faculty of Medicine, Chiang Mai University, Chiang Mai 50200, Thailand; 2 Department of Community Medicine, Faculty of Medicine, Chiang Mai University, Chiang Mai 50200, Thailand; 3 Center for Research and Development of Natural Products for Health, Chiang Mai University, Chiang Mai 50200, Thailand

**Keywords:** aging, frailty, fried’s phenotypes, frailty biomarkers, C-reactive protein, interleukin-6, cross-sectional study, Thailand

## Abstract

Thailand has officially reached the status of an “aged society” and become the developing country with the 2nd largest proportion of senior citizens in Southeast Asia. A cross-sectional study of 526 early-old community dwellers was conducted for the Fried frailty phenotype assessment, This included five indicators: Weakness, slowness, physical activity, exhaustion, and weight loss. C-reactive protein (CRP), interleukin-6 (IL-6), insulin-like growth factor-1, and CD4+:CD8+ Ratio which serve as blood-based biomarkers of frailty. The prevalence of frailty and pre-frail in this population was found to be 15% and 69.6% respectively and was higher among women than men. Frail (*n* = 58) and non-frail (*n* = 60) participants were evaluated for the associations between the frail indicators and the blood-based biomarkers. Serum levels of IL-6 and CRP from frail group were significantly elevated when compared with the non-frail counterparts (*p* = 0.044 and 0.033, respectively), and were significantly associated with the frailty status with an Odd Ratio_IL-6_ [OR] of 1.554-fold (95% confidence interval [CI], 1.229–1.966) and an OR_CRP_ of 1.011-fold (95 CI, 1.006–1.016). Decreased hand-grip strength was the only frailty indicator that was significantly associated with both inflammatory biomarkers, (OR_IL-6_ of 1.470-fold and OR_CRP_ of 1.008-fold). Our study is the first to assess the frailty status among the early-old population in Thailand. These findings will encourage general practitioners to combine frailty indicators and serum biomarkers as early detection tools for at-risk older adults to achieve the goal of healthy aging.

## 1. Introduction

For the past 25 years, the aging population in Thailand has been steadily increasing with those who are aged more than 60 years old estimated to reach over 11 million. This figure accounts for 16.7% of Thailand’s overall population [1]. This significant change has meant that Thailand has already reached the status of an aged society (a population in which the proportion of people who are aged 60 years or more is more than 10% of the total population [2]) and has become the country with the 2nd largest proportion of senior citizens among the Southeast Asia countries [3]. Aging involves physiological, psychological, and social changes that directly affect the health and well-being of older adults [4]. However, the primary challenges to setting appropriate healthcare policies and the effective management of health care systems are not entirely focused on the aging population but rather those who are “aging with frailty” [5]. Frailty is a crucial age-related clinical syndrome that is commonly defined as a state of increased vulnerability to external stressors and occurs as a consequence of the cumulative decline in many physiological systems over a lifetime [6]. It is widely accepted as a critical issue for aging populations worldwide, as it is relevant to multiple adverse outcomes including hospitalization, institutionalization, and premature mortality [7]. Moreover, frailty is believed to be a strong risk factor for many chronic diseases that are usually seen later in life, including obesity, diabetes, cardiovascular diseases, and neurodegenerative diseases [8,9]. According to the acknowledged risk of mortality in older adults, the highest mortality rate among Thailand’s population belongs to the northern area of the country. Moreover, Chiang Mai, a centralized province located within the northern part of Thailand, is currently ranked as having the 3rd highest number of aging individuals when compared with other provinces in Thailand. In addition, Lamphun Province has been recognized as being the province with the highest aging index in northern Thailand [1,3].

Despite the importance of this clinical syndrome, an internationally accepted definition of frailty has still not been reached. Several criteria for frailty have been proposed with the frailty phenotype (FP) and frailty index (FI) being the most commonly applied and validated criteria [10,11]. The frailty phenotype, also known as Fried’s definition, is operationalized as a syndrome that meets three or more of the five phenotypic criteria: Weakness as measured by low grip strength, slowness determined by slowed walking speed, a low level of physical activity, low energy or self-reported exhaustion, and unintentional weight loss [5,10]. While FI is characterized by the use of a non-fixed set of clinical conditions and diseases, estimating the amount of accumulated deficits/functional losses. Each approach (FP and FI) has its advantages and disadvantages. In our study, we are looking for a simple and effective tool (e.g., a self-report questionnaire with a limited number of items) that could be used among large populations of community-dwelling older people. Therefore, Fried’s frailty criteria seem to reflect such a tool, as our study designs have a pre-defined set of criteria made up of five phenotypes of frailty that were recorded by local clinical practitioners in community medicine. Another approach that is used to assess frailty in a clinical setting is the use of biomarkers, which are both accessible and accurate for the assessment of frailty by clinicians. Notably, many potential biomarkers of frailty include inflammatory biomarkers (C-reactive protein, interleukin 6, and tumor necrosis factor-α), immune cell biomarkers (CD4^+^/CD8^+^ T cell ratio), and endocrine biomarkers (insulin-like growth factor-1) [12,13,14].

A previous systematic review of the prevalence of frailty among community-dwelling adults aged >65 years in low-income and middle-income countries to be 10.7% (within a range of 4.0 to 59.1%) [15]. In Asia, the prevalence of frailty in Japan was found to be 16.0%, and it was 18.3% among the older adults living on the East Coast of Peninsular Malaysia [16,17]. The prevalence of frailty is known to increase with age and is more highly prevalent in people with low education levels, lower incomes, and self-rated health care systems. Additionally, the prevalence of frailty is also associated with higher rates of comorbidity [18]. Gender has been identified as another influencing factor for the prevalence of frailty, wherein it was found to be higher among women than men according to an African American cohort study involving 5317 men and women aged 65 years and older (14.4% and 7.4%, respectively) [10,19]. Currently, the prevalence of frailty among the aging population of Thailand using the frailty index revealed a high prevalence of frailty (22.1%) and could be used to predict mortality (with a hazard ratio of 2.34 with 95% CI 2.10–2.61) [20]. However, there still has not been any study involving an assessment of the frailty status using both frailty phenotypes and biomarkers among Thailand’s population, especially in the region where the highest proportion of the aging population has been identified [1]. Moreover, determination of the frailty status among the early-old population would be an effective tool for general practitioners in the field of geriatric preventative medicine and would allow doctors to detect the early signs of illness and help them prevent further aging clinical complications, along with incidences of comorbidity and/or mortality. Additionally, aging individual would be able to receive proper intervention at the most appropriate time. The purpose of this study was to determine the prevalence of frailty among community-dwelling older adults in Thailand, and to determine whether there is an association between frailty status and frailty biomarkers (inflammatory, endocrine, and immune cell biomarkers), and also to determine whether these biomarkers are relevant to any frailty phenotypes or sex differences among this population.

## 2. Methods

### 2.1. Study Design and Participants

This cross-sectional study involved the recruitment of 526 early-old members of an aging population (The “early-old population” definition is based on previously described [21,22], with the early-old population encompassing people between the ages of 65–74 years), who are community-dwelling adults that live in Pasang District in Lamphun Province and Khua Mung District in Chiang Mai Province. Both provinces are located in the northern part of Thailand. In accordance with the aging population of each region of Thailand, the northern region is recognized as having the highest proportion of aging citizens in the country. Moreover, with regard to the statistics of specific provinces, Chiang Mai Province is currently recognized as having the third largest elderly population, while Lamphun Provinces is ranked as having the highest aging index in northern Thailand [1]. For these reasons, we decided to choose these two provinces located in the northern region of Thailand as our geographical area of study. To establish a baseline, all participants underwent a detailed multidimensional health assessment that served as a screening process for the selection of frail (*n* = 79), pre-frail (*n* = 366), and non-frail (*n* = 81) participants in this study. Since this is a preliminary report on the association of frailty phenotypes and blood-based biomarkers, we selected only frail and non-frail participants in this study design. These screening processes were overseen by well-trained researchers and doctors working at the Department of Community Medicine, Faculty of Medicine, Chiang Mai University. The screening method was comprised of questionnaires, physical measurements, and a review of medical records that were held by the general practitioners working in the area. Participants were asked whether a doctor had ever told them that they had any of the following conditions: Myocardial infarction, diabetes or high blood sugar, high blood cholesterol, osteoarthritis, or osteoporosis. Symptoms of depression were assessed using a depression rating scale by 9Q assessment questionnaire presented in a Thai version with scores ranging from 0 to 27 (severe depression). Cognitive impairment was also assessed by the Mini-Mental State Examination-Thai version (MMST10) with scores ranging from 0 (impaired) to 29 [23].

**Figure 1:**
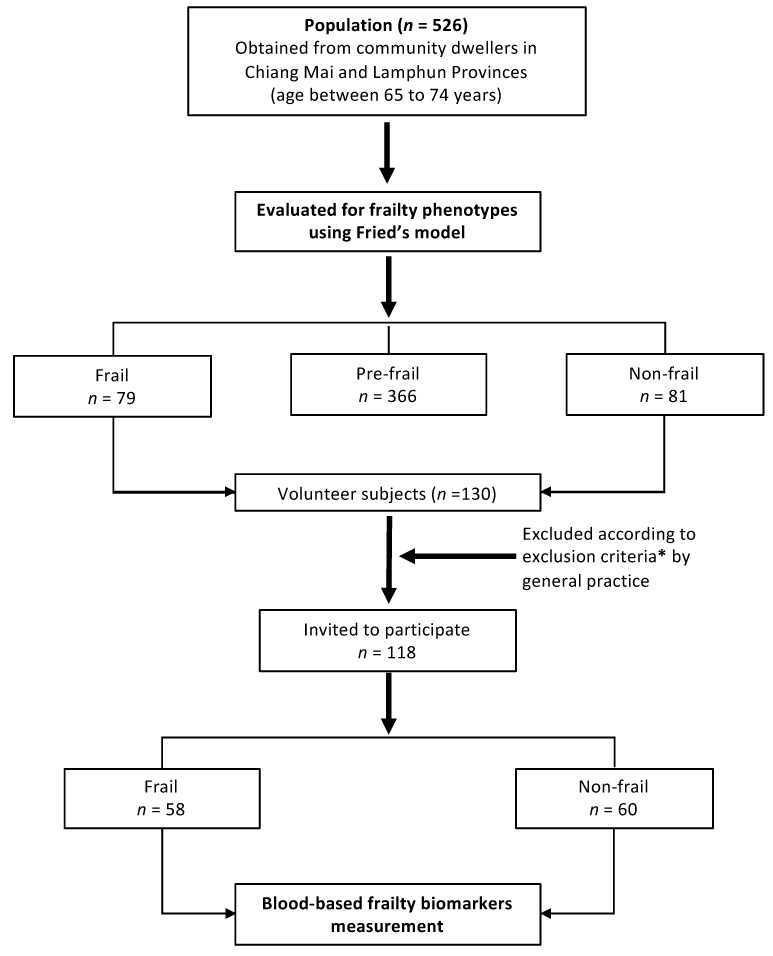
Diagram of sample selection in this study. * The exclusion criteria is when the participants had met either one or more of the following criteria; (i.) Severe locomotion problem (ii.) Ongoing major surgery or had previous undergone a major surgical procedure within the last six months, (iii.) Severe brain illness diagnosed by a general practitioners, (iv.) Severe eye or vision problems, (v.) History of cancer or tumor diagnosis, (vi.) Severe clinical illness such as severe diabetes, cardiovascular diseases, etc., and (vii.) Severe depression as assessed by a general practitioner.

The community-dwelling older adults among this population (*n* = 160: Frail; *n*= 79 and non-frail; *n* = 81) were then asked voluntarily to participate in a blood-based frailty biomarker measurement. Notably, 130 older adults were willing to participate in this measurement step. Unsuitable participants were then excluded according to the exclusion criteria (described in Figure 1). Finally, frail (*n* = 58) and non-frail (*n* = 60) older adults were recruited for evaluation of their frailty biomarkers in order to confirm the presence of an association between the frailty status and frailty biomarkers. The sample selection criteria were based on age and were sex-matched among members of the frail and non-frail groups. Sample selection was carefully conducted with regard to the generalization of the population of this study group. The sample selection diagram is presented in Figure 1.

### 2.2. Frailty Measurement

In this study, frailty was defined using Fried’s frailty phenotype criteria [10]. The phenotype was comprised of five criteria: Weight loss, exhaustion, low physical activity, weakness, and slowness. Weight loss was indicated if participants lost more than 5 kg of weight in the prior year. Exhaustion was indicated by the self-reporting of participants through the use of a questionnaire and was then calculated as an exhaustion-sum score. Low physical activity was indicated by the responses of participants to questions on the frequency with which they undertook vigorous, moderate, or mild activities. Low physical activity was indicated if a subject fell into the lowest quartile of activity as measured by the Physical Activity Scale for the Elderly (Kcal.). A slow walking speed was indicated by the amount of time a participant spent walking, which was measured by a timed session of a 4.5 m walk that was then stratified by that participant’s height and sex. Weakness was determined by grip-strength, which was measured three times on the subject’s dominant side with a digital handgrip dynamometer (T.K.K. 5401, Takei Scientific Instruments Corporation, Japan). Weakness of grip-strength was determined based on sex and body mass index (BMI) as has been described previously [10]. The highest recorded value of grip-strength was taken as the maximal value. Finally, participants were classified as “non-frail” if they met none of the criteria, “pre-frail” if they met one or two data points of the criteria, and “frail” if older individuals met three or more data points of the criteria. The questionnaires used in this study included a general health assessment and a frailty screening method are provided in the supplementary materials.

### 2.3. Blood-Based Frailty Biomarker Measurement

After an overnight fast, 15 mL of blood was drawn from the antecubital vein between 7:00 and 9:30 am. Great attention was paid to deliver blood samples as quickly as possible to the central laboratory of the Associated Medical Sciences Clinical Services Center, Chiang Mai University. EDTA plasma and aliquot of the serum was obtained and analyzed for complete blood count (CBC), the presence of liver enzymes (Aspartate aminotransferase and Alanine aminotransferase: AST and ALT, respectively), kidney function tests (Blood Urea Nitrogen:BUN and Creatinine), and a lipid profile (total cholesterol, triglycerides, HDL-Cholesterol, and LDL-Cholesterol). Plasma contained in NaF tubes was analyzed for fasting blood glucose (FBS).

Polypropylene vials containing aliquots of the serum were kept frozen at −80 °C and stored as blood samples for further use in determining Interleukin-6 (IL-6), Insulin-like growth factor-1 (IGF-1), and C-reactive protein (CRP) levels. The serum levels of these frailty biomarkers were measured with ELISA kits (IL-6; Biolegend, USA and CRP and IGF-1; Abcam, UK). For lymphocyte immunophenotyping, blood samples were analyzed by three-color direct immunofluorescence (Tri-test) flow-cytometry (Becton Dickinson FACScan Flow Cytometer) for CD4 fluorescein isothiocyanate (FITC)/CD8 phycoerythrin (PE)/CD3 peridinin chlorophyll protein (PerCP). The fluorescence-labeled antibody was obtained from BD Biosciences (Oxford, UK).

### 2.4. Statistical Analysis

All analyses were performed using SPSS 21.0 (IBM, New York, NY, USA). The Kolmogorv-Smirnov test was used to asses normality of distribution. Continuous variables with normal distribution were expressed as mean ± SD or ± standard error of mean (SE), while categorical variables were presented as numbers and percentages. Independent Student’s t-test was used to compare normally distributed continuous variables. Chi-square test or Mann-Whitney U test was used to compare categorical variables. Binary logistic regression model, which calculated the odds ratio (OR), and 95% confidence intervals were used to identify an association between the inflammatory markers and the frailty status or the frailty indicators as well as to further determine the presence of any associations of age and sex among the subjects. A *p*-value of <0.05 or <0.01 was considered statistically significant.

### 2.5. Ethical Consideration

All subjects gave their informed consent for inclusion before they participated in the study. The study was conducted in accordance with the Declaration of Helsinki, and the protocol was approved by the Ethics Committee of Faculty of Medicine, Chiang Mai University (Ethical number: COM-2561-05171).

## 3. Results

### 3.1. Assessment of Frailty Among a Population of Early-Old Community Dwellers

According to an assessment of frailty of 526 participants (Table 1), 15% of the participants were found to be frail, 69.6% were categorized as being pre-frail, and 15.4% were classified as non-frail. The mean age for the participants was 68.59 ± 2.9 years of age. In the context of gender, the prevalence of frailty among women was higher (17.1%) than among men (11.4%), as well as with the prevalence of pre-frail status (70.0% in women and 68.9% in men). The mean age of both sexes was not statistically significant (68.75 ± 3.1 in men and 68.50 ± 2.9 in women).

### 3.2. Assessment of Blood-Based Frailty Biomarkers

We further assessed the blood-based biomarkers among volunteer participants (*n* = 118), that were divided into two groups according to their frailty status (frail and non-frail). As is shown in Table 2, the mean ages of the members of the frail and non-frail groups was not statistically significant (*p* = 0.06), as was the mean value of their body mass index (*p* = 0.83). The commonly seen clinical conditions included diabetes and hypercholesterolemia among members of both the frail and non-frail groups, while osteoarthritis was commonly observed among only members of the frail group with statistical significance among the non-frail group (*p* <0.01). The percentage of current smokers or alcohol drinkers was higher in the non-frail group.

Interestingly, the hematological parameters revealed some significant differences between members of the frail and non-frail groups. Additionally, hemoglobin, red blood cell count, and hematocrit were found to be significantly lower among members of the frail group. Moreover, liver enzyme levels (both AST and ALT) in the frail group were also found to be significantly lower when compared to the non-frail group. However, the levels of these enzymes were still within the standard reference value for the population of Thailand (5–34 U/L for AST and 0–55 U/L for ALT). The lipid profile data revealed significantly lower levels of HDL-C in the frail group, but the values were still within the normal range of reference. With regard to the risk factors for heart disease and atherosclerosis as determined by either LDL:HDL ratio or TC:HDL ratio [24], it was found that members of the frail group experienced a greater degree of risk for both parameters than those of the non-frail group (with both *p*-values displaying border-line significance).

Table 3 shows the frailty phenotypes as observed in five frailty indicators according to Fried’s model. Additionally, we also reported on the categorical variables (exhaustion-sum score) and continuous variables (weight difference, grip-strength, walking speed, and physical activity) within each of the frailty indicators. All five of the frailty indicators (or variables) were found to be significantly different when compared to those of the non-frail group (*p* < 0.01 for all variables). This outcome was similar to the results of the frailty score, where the frailty status was determined for Fried’s frailty phenotypes. In this early-old population, among the blood-based frailty biomarkers that were assessed, as is shown in Table 4, greater levels of inflammatory biomarkers (CRP and IL-6) were associated with frailty status (*p* = 0.033 and 0.044 for CRP and IL-6, respectively), while the endocrine (IGF-1) and immunosenescence (CD4+:CD8+ ratio) biomarkers were not statistically different among members of the frail and non-frail groups.

### 3.3. Frailty Phenotypes and Frailty Biomarkers within the Context of Gender Differences

We further analyzed the frailty phenotypes and frailty biomarkers in men and women separately (Table 5), and the results revealed that all frailty indicators and frailty scores among members of the frail group were statistically different when compared to members of the non-frail group for both men and women. In terms of the blood-based biomarkers, we found that both inflammatory biomarkers (CRP and IL-6) were at greater levels and were associated with frailty status in both sexes, while statistical differences among other types of biomarkers were not observed.

### 3.4. Association between Frailty Phenotypes and Inflammatory Biomarkers

In order to confirm an association between the inflammatory biomarkers and the frailty status, we used the binary logistic regression method for both sexes and the comparison of gender effects. Table 6 shows the odds ratio values and 95% confidence interval of the frailty status for each of the inflammatory biomarkers. We found that a risk of frailty was associated with increased levels of both IL-6 and CRP, with the former being more associated with frailty risk (OR for IL-6 = 1.554 and OR for CRP = 1.011 with *p* < 0.01 for both biomarkers). In terms of a sex-difference context, the odd ratio value of the frailty risk to the IL-6 levels was higher in men (OR for IL-6 = 1.927 and 1.806 in men and women, respectively, with *p* < 0.05 for both sexes), while the frailty risk to the CRP levels was higher in women (OR for CRP = 1.004 and 1.011 in men and women, respectively, with *p* < 0.05 for both sexes). Although the OR for CRP is marginally related to frailty status (as seen in the ORs that are close to one, as well as the 95% CIs), the *p*-values of the ORs are still considered significant.

Finally, we further examined the association between each of frailty phenotypes, referred to as the frailty indicators and inflammatory biomarkers. Table 7 shows the odds ratio for each of the frailty indicators to levels of the inflammatory biomarkers. We found that among these frailty indicators, a decreased grip-strength (also known as weakness phenotype) was the only phenotype that was significantly associated with increased levels of inflammatory biomarkers (both IL-6 and CRP) among all participants (OR for IL-6 = 1.470 and OR for CRP = 1.008, *p* < 0.01 for both). In the context of sex-differences, decreased grip strength was significantly associated with increased levels of IL-6 in women, but not in men (OR for IL-6 = 1.075 with *p* = 0.15 and OR = 1.393 with *p* < 0.01 in men and women, respectively). On the other hand, increased levels of CRP were associated with decreased grip strength in both men and women (OR for CRP = 1.014 and 1.009 for men and women, respectively with *p* < 0.05 for both). Nevertheless, the odds ratio value of CRP was not as strong as what had been observed in IL-6 (as seen in the ORs of CRP that are very close to one as well as 95% CIs). In men, along with a decrease in grip strength, increased levels of CRP were also found to be associated with the exhaustion sum score (also known as exhaustion phenotype) and a decreased physical activity phenotype (OR = 1.002 for both phenotypes with *p* < 0.05).

## 4. Discussion

According to the United Nations statistics on the world’s aging population, the global population aged 60 years or over numbered 962 million in 2017. This number was more than twice as large as it was in 1980. The number of older persons is expected to double again by 2050 when it is projected to reach nearly 2.1 billion [25]. In the meantime, the older population of developing regions is growing much faster than in developed regions. Asia is also expected to experience a two-fold increase in the number of older persons, with the population aged 60 or over projected to increase from 549 million in 2017 to nearly 1.3 billion in 2050 [3,25]. Thailand, as a developing country, is now classified as an “aged society.” According to the statistical report published by Thailand’s National Statistical Office, the total population of Thai people in 2017 was 65.1 million with 16% of its population accounting for people aged 60 or over. These 10.3 million included 4.6 million males and 5.7 million females [3]. Population aging is driven by reductions in fertility rates and improvements in survival that occur during demographic transitions. The ife expectancy of Thai people has increased from 59 to 75 years over the last 50 years. Thus, healthcare systems and healthcare management policies for older adults are essential and should be improved upon. According to a Thai National Health Examination Survey (NHES), aging individuals with serious illnesses and/or health conditions, including joint inflammation/degeneration, emphysema, chronic lung disease, myocardial infarction, heart failure, and paralysis, and those community dwellers who live outside municipal areas, are more likely to have experienced an accidental fall and injury which has significantly affected their states of health [1,26,27].

Frailty is a geriatric syndrome that is caused by a range of multi-dimensional factors, including genetic, physical, biological, social, and environmental influences that are involved with pathogenesis [5,9]. This predictive clinical phenotype is believed to be associated with adverse health outcomes. According to Fried’s model of frailty phenotypes, frailty is reflected by a decreased level of functional reserves, impairment in multiple physiological systems, and a reduced ability to regain physiological homeostasis. It has a unique and distinct definition that differs from disability and comorbidity. Therefore, the presence of frailty in aging individuals is a significant implication that those individuals need health resources and clinical support [28]. In our study, we found that among other frailty biomarkers, the increased inflammatory biomarkers were most associated with frailty phenotypes, with a specific decrease being observed in the grip-strength phenotype. A great deal of evidence has suggested that chronic inflammation is associated with many age-associated diseases such as atherosclerosis, arthritis, cancer, diabetes, osteoporosis, and metabolic syndrome [5,29,30]. Many longitudinal studies have shown that along with aging; most individuals tend to develop a chronic low-grade pro-inflammatory status resulting from the triggering of life-long antigenic loads that impinge upon macrophage and the innate immune system. Mechanistically, during the aging process, various subtle internal and environmental inflammatory stimuli are mediated mainly by the increased circulatory levels of proinflammatory cytokines. The resulting prolong sub-chronic inflammation can generate Reactive Oxygen Species (ROS) causing both oxidative damage and eliciting an amplification of the cytokines’ release, causing a vicious cycle resulting in a chronic systemic pro-inflammatory state where tissue injury and healing mechanisms proceed simultaneously. The damage slowly accumulates asymptomatically over decades and finally results in a major determinant amomg both the aging process and the development of age-associated diseases such as frailty. These are often observed in conjunction with elevated levels of inflammatory cytokines such as IL-6 and CRP [31,32,33,34], which have been known to cause an imbalance among proinflammatory cytokines and anti-inflammatory cytokines. This state of inflammation is referred to as “inflamm-aging” and has been suggested to serve as a possible linkage between healthy aging and the pathogenesis of age-associated diseases [35,36]. Inflamm-aging is believed to be a key underlying mechanism that contributes to frailty through its detrimental effects, either directly or indirectly, on intermediary systems such as musculoskeletal, endocrine, cardiovascular, and hematological systems [5,37,38], which can result in sarcopenia, anemia, glucose intolerance, or dysregulated clotting factors. The consequences of which have been observed as frailty phenotypes and may ultimately lead to adverse health outcomes including falls, disabilities, dependencies, and death [9,39,40].

Many studies have found that inflammatory biomarkers are associated with frailty [12,41,42,43]. Many of these studies have reported that the levels of CRP and/or IL-6 have increased amoung frail older adults, as these biomarkers are considered predictive markers for incidents of frailty [44,45,46]. Likewise, in our study, we found that increased levels of inflammatory biomarkers are also associated with frailty among the early-old Thai population in the northern region of Thailand. Inflamm-aging contributes to frailty phenotypes. Given that weakness and slowed motor performance are essential features of frailty, sarcopenia is likely a key pathophysiological contributor to frailty [5,40]. As can be seen in our findings, grip-strength is associated with increased levels of inflammatory cytokines with IL-6 being the greater odds ratio. This outcome is in agreement with the findings of previous studies which found that IL-6 can contribute to frailty through its association with decreased muscle mass and muscle strength resulting in a specific weakness phenotype of frailty (decreased grip-strength) [47,48]. We also found that our frail participants revealed diminished levels of red blood cell counts and lower hematocrit and hemoglobin when compared with non-frail participants. This outcome is considered evidence of early signs of anemia as it has been reported to occur along with inflammatory biomarkers among frail older individuals [39]. Mechanistically, anemia is directly involved with increased levels of IL-6 and reveals an inverse relationship between hemoglobin and IL-6 in frail patients, with elevated levels of IL-6 directly inhibiting erythropoietin or interfering with iron metabolism [46,49,50]. Our study found a relationship between inflammatory biomarkers and the frailty status in a sex-difference context; that is, both CRP and IL-6 were associated with frailty status in men and women. The odds ratio of IL-6 in women was higher than CRP, and the odds ratio of IL-6 in men was slightly higher than in women (OR = 1.927 and 1.806, respectively).

In contrast, the odds ratio of CRP in women was slightly higher than in men (OR = 1.011 and 1.004, respectively). According to aforementioned results, we can assume that in the early-old population of northern Thailand, there was a relationship between inflammatory biomarkers and frailty status; however, the risk ratio between different sexes was quite similar. Notably, in previously published data, the relationship of frailty biomarkers in the context of sex-differences was varied and inconsistent [51]. In agreement with our results, three other previous studies found that the association between the range of immune-endocrine markers and the risk of frailty was similar among men and women [52,53,54]. However, in an English longitudinal study of aging individuals, it was found that the relationship between CRP and incidents of frailty in the population aged over 60 to 90 years (*n* = 2,146) differed significantly according to sex, with women displaying a higher risk at a ratio of 1.69 (95% CI: 1.32, 2.17) [51]. Another study found no association between the levels of CRP and incidents of frailty, even when subjects were separated by sex [53].

Another pathological process that many researchers believe contributes to frailty pathogenesis is immunosenescence, which is the phenomenon wherein adaptive immunity declines and is observed as changes in CD4+ and CD8+ T cell proportions with a CD4:CD8 ratio of less than one [55,56,57]. However, since our study has focused on the early-old population that is acknowledged as being in an early stage of aging, the aberrant in the immunosenescene biomarkers would be less likely to be observed as prominent as in the later stages of aging [12,42]. Notably, there were inconclusive results regarding the serum IGF-1 as a frailty biomarker. According to a previous study conducted among a small group of the aging population, a trend toward an inverse relationship between IGF-I and IL-6 levels in frail older adults was observed [58]. However, another published report involving a larger population demonstrated that a significant difference of serum IGF-1 levels between frail and non-frail group was not observed [52]. This outcome was similar to our findings in that the serum’s IGF-1 had no association with frailty status. Overall our data revealed that only two serum inflammatory biomarkers (IL-6 and CRP) are profoundly associated with the frailty status in a specific frailty indicator: Decreased grip strength. This might be due to the limitation of our study involving the sample size of an aging population and the inclusion of certain self-reported data (for some of the frailty indicators such as self-reported weight loss or exhaustion sum score) which could have affected the significance outcome.

## 5. Conclusions

To our knowledge, this study was the first to combine both frailty phenotypes and frailty biomarkers to assess the frailty status among Thailand’s aging population. This study is also believed to be the first report on the prevalence of frailty in northern Thailand. Through the use of an extended sample size, we would be able to positively confirm the findings for this population. However, the findings of our study support the use of biomarkers as predictors of further adverse clinical health outcomes or chronic diseases among aging individuals and can serve as an early form of detection for frailty status. However, full validation of these biomarkers should be confirmed through further studies. We hope that this study will bring about better healthcare policies for the aging population in Thailand and help the government to realize the importance of elderly care as Thailand begins to enter the era of an aging society.

## Figures and Tables

**Table 1:** Frailty prevalence (operationalized by Fried’s model) among early-old community dwellers population in the northern part of Thailand (*n* = 526).

Gender	Prevalence %, (95% CI)		
	Frail ^a^	Pre-Frail ^b^	Non-Frail ^c^
Overall (*n* = 526)	15.0 (12.1, 18.4)	69.6 (65.5, 73.5)	15.4 (12.4, 18.8)
Men (*n* = 193)	11.4 (7.3, 16.7)	68.9 (61.9, 75.4)	19.7 (14.3, 26.0)
Women (*n* = 333)	17.1 (13.2, 21.6)	70.0 (64.7, 74.8)	12.9 (9.5, 17.0)

**^a^** Frail indicated if the subject met 3 or more of the criteria. **^b^** Pre-Frail indicated if the subject met 1 or 2 of the criteria. **^c^** Non-Frail indicated if the subject met none of the frail phenotypic criteria.

**Table 2:** Characteristics of volunteer participants according to frailty status (*n* = 118).

Data	Frail (*n* = 58)	Non-Frail (*n* = 60)	*p*-Value
**Sociodemographic factors** Age, mean ± SD	68.9 ± 2.7	68.1 ± 2.5	0.06
Female, *n* (%)	41 (70.7)	35 (58.3)	0.23
Married/living with partner, *n* (%)	33 (56.9)	41 (68.3)	0.27
Lifestyle characteristics			
Smoking at present, *n* (%)	4 (6.9)	7 (11.7)	0.57
Alcohol at present, *n* (%)	3 (5.2)	11 (18.3)	0.05
**Clinical health data**			
Body mass index (BMI), mean ± SD	22.1 ± 4.1	22.2 ± 2.8	0.83
**History of illness, *n* (%)**			
Cardiovascular diseases	4 (6.9)	0 (0)	0.12
Diabetes	10 (17.2)	9 (15.0)	0.94
** Osteoarthritis	16 (27.6)	4 (6.7)	<0.01
Asthma or Chronic lung diseases	1 (1.7)	1 (1.7)	1.00
Hypercholesterolemia	12 (20.7)	10 (16.7)	0.75
Osteoporosis	0 (0)	2 (3.3)	0.49
Depression score ≥7, *n* (%)	8 (13.8)	3 (5.0)	0.19
* Cognitive function, mean ± SD	23.1 ± 3.9	24.9 ± 3.7	0.02
**Hematological data, mean ± SD**			
* Hemoglobin (g/dL)	12.5 ± 1.6	13.3 ± 1.6	0.01
* Red blood cell count (10^6^/µL)	4.8 ± 0.8	5.1 ± 0.5	<0.01
* Hematocrit (%)	39.2 ± 4.5	41.4 ± 4.0	<0.01
White blood cell count (10^3^/µL)	7.2 ± 1.8	6.9 ± 1.7	0.41
Neutrophil (%)	55.2 ± 8.8	52.9 ± 10.3	0.17
Lymphocyte (%)	31.5 ± 8.2	33.3 ± 8.5	0.21
Monocyte (%)	7.7 ± 2.0	7.7 ± 1.9	0.97
Blood urea nitrogrn (mg/dL)	14.5 ± 4.6	13.9 ± 4.6	0.44
Creatinine (mg/dL)	0.9 ± 0.3	0.9 ± 0.2	0.68
** AST (U/L)	20.4 ± 6.0	24.7 ± 10.8	<0.01
** ALT (U/L)	15.8 ± 7.0	20.9 ± 12.7	<0.01
Fasting blood glucose (mg/dL)	101.0 ± 26.5	105.5 ± 27.7	0.35
**Lipid profile, mean ± SD**			
Total cholesterol	200.8 ± 50.9	203.6 ± 38.1	0.73
Triglycerides (mg/dL)	142.2 ± 79.0	125.0 ± 57.7	0.08
** HDL-C (mg/dL)	49.4 ± 10.4	54.1 ± 11.4	<0.01
LDL-C (mg/dL)	123.0 ± 42.7	124.5 ± 32.4	0.83
LDL-c/ HDL-c Ratio	2.56 ± 0.9	2.37 ± 0.7	0.09
TC/ HDL-c Ratio	4.19 ± 1.2	3.88 ± 0.9	0.05

* 0.01< *p* < 0.05, ** *p* < 0.01 (Statistical significance using Chi-square test for categorical data and independent t-test for continuous data).

**Table 3:** Comparisons of frailty indicators between the frail and the non-frail phenotype.

Parameters	Frail (*n* = 58)	Non-Frail (*n* = 60)	*p*-Value
**Frailty indicators**			
**Self-reported weight loss, *n* (%)**	18 (31.0)	0 (0)	-
** Weight difference (∆w, kg)	−2.23 ± 4.3	0.55 ± 1.7	<0.01
**Self-reported exhaustion, n (%)**	40 (69.0)	0 (0)	-
** Exhaustion sum score	2.14 ± 1.6	0.18 ± 0.4	<0.01
Weakness, *n* (%)	50 (86.2)	0 (0)	-
** Grip strength, kg	15.89 ± 5.69	25.76 ± 7.09	<0.01
**Slow walking speed, *n* (%)**	37 (63.8)	0 (0)	-
** Walking speed, s	7.28 ± 1.98	4.30 ± 0.80	<0.01
**Decreased activity, *n* (%)**	50 (86.2)	0 (0)	-
** Physical activity, Kcal	215.3 ± 470.1	1192.1 ± 1397.7	< 0.01
** Frailty score	3.36 ± 0.52	0	< 0.01

* 0.01< *p* < 0.05, ** *p* < 0.01 (Data are represented as mean ± SD and statistical significance using the independent t-test for continuous data and Mann-Whitney U test for categorical data).

**Table 4:** Comparisons of blood-based biomarkers between the frail and the non-frail phenotype.

Biomarkers	Frail (*n* = 58)	Non-Frail (*n* = 60)	p-Value
**Inflammatory Biomarkers**			
* C-reactive protein, mg/L	0.67 ± 0.13	0.40 ± 0.05	0.033
* Interleukin-6, pg/mL	20.01 ± 8.14	5.80 ± 0.55	0.044
**Endocrine biomarkers**			
Insulin-like growth factor-1, ng/mL	29.96 ± 0.85	28.47 ± 0.80	0.103
**Immunosenescence biomarkers**			
% CD4+ T cell	36.62 ± 1.07	33.88 ± 1.07	0.073
% CD8+ T cell	20.02 ± 0.96	19.67 ± 0.92	0.794
**CD4:CD8 Ratio**	2.13 ± 0.13	2.06 ± 1.19	0.735

* 0.01< *p* < 0.05, ** *p* < 0.01 (Data are represented as mean ± SE and Statistical significance using the independent t-test).

**Table 5:** Gender effects on comparisons of frailty indicators and blood-based biomarkers between the frail and the non-frail phenotype [men (*n* = 42) and women (*n* = 76)].

Parameters	Men			Women		
	Frail (*n* = 17)	Non-Frail (*n* = 25)	*p*-Value	Frail (*n* = 41)	Non-Frail (*n* = 35)	*p*-Value
**Frailty indicators, n (%)** **Self-reported weight loss**	6 (35.3)	0	-	12 (29.3)	0	-
** Weight difference (∆w, kg)	−3.09 ± 4.0	0.59 ± 1.7	<0.01	−1.88 ± 4.4	0.53 ± 1.7	<0.01
**Self-reported exhaustion**	13 (76.5)	0	-	27 (65.9)	0	-
** Exhaustion sum score	2.65 ± 1.8	0.12 ± 0.3	<0.01	1.93 ± 1.4	0.23 ± 0.4	<0.01
**Weakness**	14 (82.4)	0	-	36 (87.8)	0	-
** Grip strength, kg	20.6 ± 5.6	32.6 ± 5.7	<0.01	13.9 ± 4.5	20.9 ± 2.3	<0.01
**Slow walking speed**	6 (35.3)	0	-	31 (75.6)	0	-
** Walking speed, s	6.2 ± 1.4	4.0 ± 0.7	<0.01	7.7 ± 2.0	4.5 ± 0.8	<0.01
**Decreased activity, kcal**	14 (82.4)	0	-	36 (87.8)	0	-
** Physical activity, Kcal	363.8 ± 755.4	1481.2 ± 1382.9	<0.01	153.7 ± 273.0	985.5 ± 1391.0	<0.01
** Frailty score, mean ± SD	3.1 ± 0.3	0	<0.01	3.5 ± 0.6	0	<0.01
**Frailty biomarkers, mean ± SE**						
** C-reactive protein, mg/L	0.47 ± 0.1	0.24 ± 0.04	0.005	0.75 ± 0.19	0.51 ± 0.07	0.001
* Interleukin-6, pg/mL	10.44 ± 3.7	5.69 ± 0.8	0.033	23.98 ± 11.4	5.88 ± 0.8	0.034
IGF-1, ng/mL	32.57 ± 1.0	29.57 ± 1.2	0.072	28.88 ± 1.1	27.69 ± 1.0	0.432
% CD4 + T cell	31.64 ± 8.1	36.86 ± 7.2	0.023*	34.66 ± 8.6	36.74 ± 9.6	0.309
% CD8 + T cell	18.63 ± 10.4	19.20 ± 7.1	0.822	21.11 ± 7.3	19.47 ± 6.1	0.286
CD4:CD8 Ratio	2.19 ± 1.2	2.27 ± 1.2	0.834	1.90 ± 1.0	2.14 ± 1.1	0.315

* 0.01< *p* < 0.05, ** *p* < 0.01 (Data are represented as mean ± SD or ± SE and statistical significance using Mann-Whitney U test for categorical data (Exhaustion sum score) and the independent t-test for continuous data).

**Table 6:** Relationship of serum inflammatory biomarkers and the frail and the non-frail phenotype using binary linear regression analysis (odds ratios) with the comparison on gender effects.

Frailty category (non-frail, frail)	Interleukin-6		C-reactive Protein	
	OR (95% CI)	*p*-Value	OR (95% CI)	*p*-Value
** Overall	1.554 (1.229, 1.966)	<0.001	1.011 (1.006, 1.016)	<0.001
* Men	1.927 (1.138, 3.262)	0.015	1.004 (1.001, 1.007)	0.020
** Women	1.806 (1.215, 2.683)	0.003	1.011 (1.003, 1.018)	0.005

* 0.01< *p* < 0.05, ** *p* < 0.01 (Statistical significance using binary linear regression analysis).

**Table 7:** Relationship of serum inflammatory biomarkers and the frailty indicators using binary linear regression analysis (odds ratios) with the comparison of gender effects.

Frailty indicators	Interleukin-6		C-reactive Protein	
	OR (95% CI)	*p*-Value	OR (95% CI)	*p*-Value
**Overall**				
Weight difference (∆w, kg)	0.996 (0.976, 1.016)	0.683	1.000 (0.999, 1.001)	0.530
Exhaustion sum score	1.006 (0.995, 1.016)	0.278	1.000 (0.999, 1.000)	0.512
** Grip strength (kg)	1.470 (1.200, 1.801)	<0.01 **	1.008 (1.004, 1.011)	<0.01 **
Walking speed (s)	1.017 (0.993, 1.042)	0.165	1.001 (1.000, 1.001)	0.074
Physical activity (Kcal)	1.001 (0.993, 1.009)	0.797	1.001 (1.000, 1.002)	0.054
**Men**				
Weight difference (∆w, kg)	1.038 (0.973, 1.107)	0.256	1.000 (0.997, 1.002)	0.850
Exhaustion sum score	1.061 (0.979, 1.151)	0.149	1.002 (1.000, 1.005)	0.037 *
Grip strength (kg)	1.075 (0.974, 1.186)	0.150	1.014 (1.003, 1.026)	0.012 *
Walking speed (s)	0.859 (0.624, 1.183)	0.352	1.001 (0.999, 1.003)	0.417
Physical activity (Kcal)	1.089 (0.969, 1.225)	0.153	1.002 (1.000, 1.005)	0.044 *
**Women**				
Weight difference (∆w, kg)	0.989 (0.948, 1.032)	0.616	1.000 (0.999, 1.001)	0.523
Exhaustion sum score	1.004 (0.995, 1.014)	0.368	0.999 (0.998, 1.000)	0.195
** Grip strength (kg)	1.393 (1.088, 1.782)	<0.01 **	1.009 (1.002, 1.015)	<0.01 **
Walking speed (s)	1.028 (0.983, 1.075)	0.225	1.000 (1.000, 1.001)	0.197
Physical activity (Kcal)	0.999 (0.991, 1.008)	0.888	1.000 (1.000, 1.001)	0.182

* 0.01< *p* < 0.05, ** *p* < 0.01 (Statistical significance using binary linear regression analysis).

## Supplementary Materials

The following are available online at https://www.mdpi.com/1660-4601/16/18/3457/s1, Questionnaire form: Questionnaire Form and Frailty Screening Method.

## References

[1] National Statistical Office (2018). Report on the 2017 Survey of the Older Persons in Thailand.

[2] Gavrilov L.A., Heuveline P. (2003). Aging of population. Encycl. Popul..

[3] Institute for Population and Social Research M.U. (2015). Situation of Thai Elderly 2015.

[4] Siriwardhana D.D., Weerasinghe M.C., Rait G., Falcaro M., Scholes S., Walters K.R. (2019). Prevalence of frailty in rural community-dwelling older adults in Kegalle district of Sri Lanka: A population-based cross-sectional study. BMJ Open.

[5] Chen X., Mao G., Leng S.X. (2014). Frailty syndrome: An overview. Clin. Interv. Aging.

[6] Hubbard R.E., Woodhouse K.W. (2010). Frailty, inflammation and the elderly. Biogerontology.

[7] Fried L.P., Ferrucci L., Darer J., Williamson J.D., Anderson G. (2004). Untangling the concepts of disability, frailty, and comorbidity: Implications for improved targeting and care. J. Gerontol. Ser. A Biol. Sci. Med. Sci..

[8] Bektas A., Schurman S.H., Sen R., Ferrucci L. (2017). Human T cell immunosenescence and inflammation in aging. J. Leukoc. Biol..

[9] Wou F., Conroy S. (2013). The frailty syndrome. Medicine.

[10] Fried L.P., Tangen C.M., Walston J., Newman A.B., Hirsch C., Gottdiener J., Seeman T., Tracy R., Kop W.J., Burke G. (2001). Frailty in older adults: Evidence for a phenotype. J. Gerontol. Ser. A Biol. Sci. Med. Sci..

[11] Rockwood K., Song X., MacKnight C., Bergman H., Hogan D.B., McDowell I., Mitnitski A. (2005). A global clinical measure of fitness and frailty in elderly people. CMAJ.

[12] Martin-Ruiz C., Jagger C., Kingston A., Collerton J., Catt M., Davies K., Dunn M., Hilkens C., Keavney B., Pearce S.H. (2011). Assessment of a large panel of candidate biomarkers of ageing in the Newcastle 85+ study. Mech. Ageing Dev..

[13] Wagner K.H., Cameron-Smith D., Wessner B., Franzke B. (2016). Biomarkers of aging: From function to molecular biology. Nutrients.

[14] Xia X., Chen W., McDermott J., Han J.D.J. (2017). Molecular and phenotypic biomarkers of aging. F1000Research.

[15] Siriwardhana D.D., Hardoon S., Rait G., Weerasinghe M.C., Walters K.R. (2018). Prevalence of frailty and prefrailty among community-dwelling older adults in low-income and middle-income countries: A systematic review and meta-analysis. BMJ Open.

[16] Doba N., Tokuda Y., Goldstein N.E., Kushiro T., Hinohara S. (2012). A pilot trial to predict frailty syndrome: The Japanese Health Research Volunteer Study. Exp. Gerontol..

[17] Mohd Hamidin F.A., Adznam S.N.A., Ibrahim Z., Chan Y.M., Abdul Aziz N.H. (2018). Prevalence of frailty syndrome and its associated factors among community-dwelling elderly in East Coast of Peninsular Malaysia. Sage Open Med..

[18] Buckinx F., Rolland Y., Reginster J.Y., Ricour C., Petermans J., Bruyère O. (2015). Burden of frailty in the elderly population: Perspectives for a public health challenge. Arch. Public Health.

[19] Xue Q.L. (2011). The frailty syndrome: Definition and natural history. Clin. Geriatr. Med..

[20] Srinonprasert V., Chalermsri C., Aekplakorn W. (2018). Frailty index to predict all-cause mortality in Thai community-dwelling older population: A result from a National Health Examination Survey cohort. Arch. Gerontol. Geriatr..

[21] Kingston A., Comas-Herrera A., Jagger C. (2018). Forecasting the care needs of the older population in England over the next 20 years: Estimates from the Population Ageing and Care Simulation (PACSim) modelling study. Lancet Public Health.

[22] House J.S., Kessler R.C., Herzog A.R., Mero R.P., Kinney A.M., Breslow M.J. (1990). Age, socioeconomic status, and health. Milbank Q..

[23] Silpakit O., Silpakit C., Pukdeenaul P. (2007). A comparison study of cognitive impairment screening tools: CDT, IQCODE VS MMSE. Siriraj. Med. J..

[24] Lemieux I., Lamarche B., Couillard C., Pascot A., Cantin B., Bergeron J., Dagenais G.R., Després J.P. (2001). Total cholesterol/HDL cholesterol ratio vs LDL cholesterol/HDL cholesterol ratio as indices of ischemic heart disease risk in men: The Quebec Cardiovascular Study. Arch. Intern. Med..

[25] United Nation The Department of Economic and Social Affairs (2017). Population Division. World Population Ageing 2017.

[26] Hongthong D., Somrongthong R. (2015). Factors influencing the Quality of Life (Qol) among Thai older people in a rural area of Thailand. Iran. J. Public Health.

[27] Manasatchakun P., Roxberg Å., Asp M. (2018). Conceptions of healthy aging held by relatives of older persons in Isan-Thai culture: A phenomenographic study. J. Aging Res..

[28] Knodel J., Chayovan N. (1997). Family support and living arrangements of Thai elderly. Asia Pac. Popul. J..

[29] Rockwood K., Fox R.A., Stolee P., Robertson D., Beattie B.L. (1994). Frailty in elderly people: An evolving concept. CMAJ Can. Med. Assoc. J..

[30] Fulop T., Larbi A., Witkowski J.M., McElhaney J., Loeb M., Mitnitski A., Pawelec G. (2010). Aging, frailty and age-related diseases. Biogerontology.

[31] Lang P.O., Michel J.P., Zekry D. (2009). Frailty syndrome: A transitional state in a dynamic process. Gerontology.

[32] Salvioli S., Capri M., Valensin S., Tieri P., Monti D., Ottaviani E., Franceschi C. (2006). Inflamm-aging, cytokines and aging: State of the art, new hypotheses on the role of mitochondria and new perspectives from systems biology. Curr. Pharm. Des..

[33] Franceschi C., Capri M., Garagnani P., Ostan R., Santoro A., Monti D., Salvioli S. (2018). Inflammaging. Handbook of Immunosenescence: Basic Understanding and Clinical Implications.

[34] Limtrakul P., Yodkeeree S., Pitchakarn P., Punfa W. (2015). Suppression of inflammatory responses by black rice extract in RAW 264.7 macrophage cells via downregulation of NF-kB and AP-1 signaling pathways. Asian Pac. J. Cancer Prev..

[35] Franceschi C., Bonafè M., Valensin S., Olivieri F., De Luca M., Ottaviani E., De Benedictis G. (2000). Inflamm-aging: An evolutionary perspective on immunosenescence. Ann. N. Y. Acad. Sci..

[36] Miquel J. (2009). An update of the oxidation-inflammation theory of aging: The involvement of the immune system in oxi-inflamm-aging. Curr. Pharm. Des..

[37] Fulop T., Larbi A., Dupuis G., Le Page A., Frost E.H., Cohen A.A., Witkowski J.M., Franceschi C. (2018). Immunosenescence and inflamm-aging as two sides of the same coin: Friends or foes?. Front. Immunol..

[38] Ferrucci L., Fabbri E. (2018). Inflammageing: Chronic inflammation in ageing, cardiovascular disease, and frailty. Nat. Rev. Cardiol..

[39] Röhrig G. (2016). Anemia in the frail, elderly patient. Clin. Interv. Aging.

[40] Morley J.E., Malmstrom T.K. (2013). Frailty, sarcopenia, and hormones. Endocrinol. Metab. Clin..

[41] Leng S.X., Xue Q.L., Tian J., Walston J.D., Fried L.P. (2007). Inflammation and frailty in older women. J. Am. Geriatr. Soc..

[42] Collerton J., Martin-Ruiz C., Davies K., Hilkens C.M., Isaacs J., Kolenda C., Parker C., Dunn M., Catt M., Jagger C. (2012). Frailty and the role of inflammation, immunosenescence and cellular ageing in the very old: Cross-sectional findings from the Newcastle 85+ Study. Mech. Ageing Dev..

[43] Yang Y., Hao Q., Flaherty J.H., Cao L., Zhou J., Su L., Shen Y., Dong B. (2018). Comparison of procalcitonin, a potentially new inflammatory biomarker of frailty, to interleukin-6 and C-reactive protein among older Chinese hospitalized patients. Aging Clin. Exp. Res..

[44] De Martinis M., Franceschi C., Monti D., Ginaldi L. (2006). Inflammation markers predicting frailty and mortality in the elderly. Exp. Mol. Pathol..

[45] Hubbard R.E., O’Mahony M.S., Savva G.M., Calver B.L., Woodhouse K.W. (2009). Inflammation and frailty measures in older people. J. Cell. Mol. Med..

[46] Darvin K., Randolph A., Ovalles S., Halade D., Breeding L., Richardson A., Espinoza S.E. (2013). Plasma protein biomarkers of the geriatric syndrome of frailty. J. Gerontol. Ser. A Biomed. Sci. Med. Sci..

[47] Pereira L.S.M., Narciso F.M.S., Oliveira D.M.G., Coelho F.M., de Souza D.D.G., Dias R.C. (2009). Correlation between manual muscle strength and interleukin-6 (IL-6) plasma levels in elderly community-dwelling women. Arch. Gerontol. Geriatr..

[48] Legrand D., Adriaensen W., Vaes B., Matheï C., Wallemacq P., Degryse J. (2013). The relationship between grip strength and muscle mass (MM), inflammatory biomarkers and physical performance in community-dwelling very old persons. Arch. Gerontol. Geriatr..

[49] Leng S., Chaves P., Koenig K., Walston J. (2002). Serum interleukin-6 and hemoglobin as physiological correlates in the geriatric syndrome of frailty: A pilot study. J. Am. Geriatr. Soc..

[50] Ershler W.B. (2003). Biological interactions of aging and anemia: A focus on cytokines. J. Am. Geriatr. Soc..

[51] Gale C.R., Baylis D., Cooper C., Sayer A.A. (2013). Inflammatory markers and incident frailty in men and women: The English Longitudinal Study of Ageing. Age.

[52] Puts M.T., Visser M., Twisk J.W., Deeg D.J., Lips P. (2005). Endocrine and inflammatory markers as predictors of frailty. Clin. Endocrinol..

[53] Baylis D., Bartlett D.B., Syddall H.E., Ntani G., Gale C.R., Cooper C., Lord J.M., Sayer A.A. (2013). Immune-endocrine biomarkers as predictors of frailty and mortality: A 10-year longitudinal study in community-dwelling older people. Age.

[54] Barzilay J.I., Blaum C., Moore T., Xue Q.L., Hirsch C.H., Walston J.D., Fried L.P. (2007). Insulin resistance and inflammation as precursors of frailty: The Cardiovascular Health Study. Arch. Intern. Med..

[55] Gruver A., Hudson L., Sempowski G. (2007). Immunosenescence of ageing. J. Pathol. A J. Pathol. Soc. Great Br. Irel..

[56] Pawelec G., Adibzadeh M., Pohla H., Schaudt K. (1995). Immunosenescence: Ageing of the immune system. Immunol. Today.

[57] Correa B.L., Ornaghi A.P., Muller G.C., Engroff P., Lopes R.P., da Silva Filho I.G., Bosch J.A., Bonorino C., Bauer M.E. (2014). The inverted CD4: CD8 ratio is associated with cytomegalovirus, poor cognitive and functional states in older adults. Neuroimmunomodulation.

[58] Leng S.X., Cappola A.R., Andersen R.E., Blackman M.R., Koenig K., Blair M., Walston J.D. (2004). Serum levels of insulin-like growth factor-I (IGF-I) and dehydroepiandrosterone sulfate (DHEA-S), and their relationships with serum interleukin-6, in the geriatric syndrome of frailty. Aging Clin. Exp. Res..

