# Supplementary material for: The Association between Frailty Indicators and Blood-Based Biomarkers in Early-Old Community Dwellers of Thailand
Source: Int J Environ Res Public Health. 2019 Sep 17;16(18):3457. doi: 10.3390/ijerph16183457 (PMC6765843; doi:10.3390/ijerph16183457)
Supplement: Supplementary file 1 [file ijerph-16-03457-s001.pdf]

Subject NO. .... **Location**: ( ) CM ( ) LP

Questionnaire Form and Frailty Screening Method: Project NO. PRP6005021700

Instructions: Ask participant the following statements and fill out the form by placing a check mark (✓) in the appropriate box (☐)

Section 1 : General information and Health assessment

1. Age .... years

2. Sex ☐ 1. Male ☐ 2. Female

3. Marital status ☐ 1. Single ☐ 2. Married/ living with partner

☐ 3. Divorced ☐ 4. Separated ☐ 5. Widowed

4. Do you now, or have you ever had, any of the following diseases or medical problems that was being diagnosed by physicians? (Can be checked (✓) more than one)

1. Cancer/Tumor	☐ 1. Y; Location	☐ 0. N
2. High blood pressure	☐ 1. Y	☐ 0. N
3. Cardiovascular diseases (MI, Angina, Heart failure, etc.)	☐ 1. N	☐ 0. N
4. Diabetes	☐ 1. Y	☐ 0. N
5. Hypercholesterolemia	☐ 1. N	☐ 0. N
6. Asthma or Chronic lung disease	☐ 1. Y	☐ 0. N
7. Osteoarthritis	☐ 1. Y	☐ 0. N
8. Osteoporosis	☐ 1. Y	☐ 0. N
9. Stroke	☐ 1. Y	☐ 0. N
10. Others	☐ 1. Y; Please specify........................	☐ 0. N

5. Do you now, or have you ever had drinking an alcohol?

☐ 1. Y; At present ☐ 2. Y; In the past ☐ 3. N; Never

6. Do you now, or have you ever had Smoking?

☐ 1. Y; At present ☐ 2. Y; In the past ☐ 3. N; Never

7. Depression symptom screening (2Q depression rating scale)

During the last month, have you often……….?

(If checked N for both 2Q questions, skip 9Q assessment and move to section 2)

...1 been bothered by feeling down, depressed or hopeless ?	☐ 1.Y	☐ 0. N
...2 been bothered by having little interest or pleasure in doing things ?	☐ 1.Y	☐ 0. N

(Answering yes to either of these questions is considered a positive test result, warranting further 9Q assessment)

8. 9Q depression rating scale questionnaire

Over the ***last 2 weeks***, how often have you been bothered by any of the following problems?	Not at all	Several days	More than half the days	Nearly everyday
1. Little interest or pleasure in doing things	0	1	2	3
2. Feeling down, depressed, or hopeless	0	1	2	3
3. Trouble falling/staying asleep, sleeping to much	0	1	2	3
4. Feeling tried or having little energy	0	1	2	3
5. Poor appetite or overeating	0	1	2	3
6. Feeling bad about yourself or that you a failure or have let yourself or family down	0	1	2	3
7. Trouble concentrating on things, such as reading the newspaper or watching television	0	1	2	3
8. Moving or speaking so slowly that other people could have noticed. Or the opposite; being so fidgety or restless that you have been moving around a lot more than usual	0	1	2	3
9. Thoughts that you would be better off dead or of hurting yourself in some way	0	1	2	3

Total 9Q score .... Points, Interpretation of 9Q rating scale;

☐ No or very mild depressive symptoms (<7 points)

☐ Mild Depression (7-12 Points)

☐ Moderate Depression (13-18 Points)

☐ Severe Depression (≥ 19 Points)

*** **If severe depression (≥ 19 Points) exclude from the study**

☐ 1. Proceed ☐ 0. Excluded from the study

+++

Section 2 : General physical examination (examined by physicians)

1. Weight ……………………. kg.

2. Height ……………………. cm. Calculated BMI = ... (kg/m^2)

3. Heart rate ……………………. bpm.

4. Blood pressure ……………………. mmHg.

Section 3: MMST10; Mini-Mental State Examination-Thai version

Instructions: Ask the questions in the order listed. Score one point for each correct response within each question or activity

Educational levels of the participants:

☐ 1. Lower than primary school/ cannot read or write in Thai ☐ 2. Primary school education

☐ 3. Higher than primary school education

(For subject who categorised in non-education; do not ask the question no.4, 9 and 10)

1. Orientation for time (5 points) **(Total .... points)**

1.1 What is the date today? ☐ 1. Correct ☐ 0. Incorrect

1.2 What is the day of the week? ☐ 1. Correct ☐ 0. Incorrect

1.3 What is the month? ☐ 1. Correct ☐ 0. Incorrect

1.4 What is the year? ☐ 1. Correct ☐ 0. Incorrect

1.5 What is the season? ☐ 1. Correct ☐ 0. Incorrect

2. Orientation for place (5 points) **(Total .... points)**

2.1 Where are we now: no.of the address? ☐ 1. Correct ☐ 0. Incorrect

2.2 Where are we now: name of the road/area ☐ 1. Correct ☐ 0. Incorrect

2.3 Where are we now: district/sub-district? ☐ 1. Correct ☐ 0. Incorrect

2.4 Where are we now: town/city/province? ☐ 1. Correct ☐ 0. Incorrect

2.5 Where are we now: region part? ☐ 1. Correct ☐ 0. Incorrect

3. Registration The examiner names three unrelated objects clearly and slowly, then asks the patient to name all three of them. The patient's response is used for scoring.

The examiner repeats them until patient learns all of them, if possible. (3 Points) **(Total .... points)**

4. Attention/Calculation (5 points, 1 point/correct answer) **(Total .... points)**

- "I would like you to count backward from 100 by sevens"

.... (93) ...... (86) ...... (79) ...... (72) ...... (65)

- Alternative question – "Spell MANOW (Lemon in Thai language) backwards"

.... (ว/W) ...... (า/O) ...... (น/N) ...... (ะ/A) ...... (ม/M)

5. Recall "Earlier I told you the names of three things. Can you tell me what those were?" (3 points) **(Total .... points)**

6. Naming Show the patient two simple objects, such as a glasses and a belt, and ask the patient to name them.

(2 points) **(Total .... points)**

7. Repetition "Repeat the phrase: **ใคร ใคร่ ขาย ไข่ ไก่ (who want to sell the chicken egg; Thai phrase)**"

(1 points) **(Total .... points)**

8. Verbal command Take the paper in your right hand, fold it in half, and put it on the floor."

(The examiner gives the patient a piece of blank paper.)

(1 point/instruction: maximun 3 points) **(Total .... points)**

9. Written command "Please read this and do what it says." (Written instruction is "Open your mouth.")

(1 point) **(Total .... points)**

10. Writing "Make up and write a sentence about anything." (This sentence must contain a noun and a verb)

(1 point) **(Total .... points)**

Interpretation of the MMST10: Suspected cognitive impairment ☐1. Yes ☐0. No

Interpretation (based on educational-levels)	Score	
	Total	Cut off score
Abnormal for "non-education (cannot read or write)"	22<br>(Do not ask the question No. 4, 9, 10)	**≤ 14**
Abnormal for "primary school education"	29	**≤ 17**
Abnormal for "higher than primary school education"	29	**≤ 22**

Section 4: Screening method for frail and non-frail participant (Fried's phenotype criteria)

1. Weight loss phenotype

Weight **in the past year** …………………… kg.

Weight **in the current year** ………………….... kg.

(If the participant cannot remember the past year's weight: use the approximate self-reported weight loss)

"The participant has loss/or gain weight for .... kg. / for the past year"

Interpretation Weight loss more than 4.5 kg. ☐ 1.Yes ☐ 2. No

2.Exhaustion phenotype

Based on 2 questions adapted from Center for Epidemiological Studies Depression (CES-D) Scale

**Question / Scoring**	0 = Fatigue or exhaution felt rarely or not at all (less than one day in the past week)	1 = Fatigue or exhaution somewhat felt at the time (1-2 days in the past week)	2 = Fatigue or exhaustion frequently felt at the time (3-4 days in the past week)	3 = Fatigue or exhaution felt most of the time (more than 4 days in the past week)
2.1 "How often **in the past week that** you felt everything you did was an effort"				
2.2 "How often **in the past week** that you felt could not get going doing everything"				

Interpretation Self-reported **Exhaustion (Exhaustion sum score)**

☐1.Yes (More than 2 points) ☐ 2. No (0-1 point)

3.Low physical activity phenotype: Global Physical Activity Questionnaire (GPAQ)

Question		Answer	Code
**Physical activities at work**			
1	Does your work involve **vigorous-intensity activity** that causes large increases in breathing or heart rate like (carrying or lifting heavy loads, digging or construction work) for at least 10 minutes continuously?	☐1. Yes<br>☐2. No<br>*(If No, go to* P4*)*	P1
2	In a typical week, on how many days do you do vigorous-intensity activities as part of your work?	Days a week	P2
3	How much time do you spend doing vigorous-intensity activities at work on a typical day?	…..hrs…...mins	P3 (a-b)
4	Does your work involve **moderate-intensity** activity that causes small increases in breathing or heart rate such as brisk walking (or carrying loads) for at least 10 minutes continuously?	☐1. Yes<br>☐2. No<br>*(If No, go to* P7*)*	P4
5	In a typical week, on how many days do you do **moderate-intensity** activities as part of your work?	Days a week	P5
6	How much time do you spend doing **moderate-intensity** activities at work on a typical day?	…..hrs…...mins	P6 (a-b)
**Question about the usual way you travel to and from places**			
The next questions exclude the physical activities at work that you have already mentioned. Now i would like to ask you about the usual way you travel to and from places. For example to work, for shopping, to market, to place of worship.			
7	Do you walk or use a bicycle (pedal cycle) for at least 10 minutes continuously to get to and from places?	☐1. Yes<br>☐2. No<br>*(If No, go to* P10*)*	P7
8	In a typical week, on how many days do you walk or bicycle for at least 10 minutes continuously to get to and from places?	Days a week	P8
9	How much time do you spend walking or bicycling for travel on a typical day?	…..hrs…...mins	P9(a-b
**Questions about sports, fitness and recreational activities**			
The next questions exclude the work and transport activities that you have already mentioned.<br>Now i would like to ask you about sports, fitness and recreational activities (leisure)			
10	Does you do any **vigorous-intensity** sports, fitness or recreational (leisure) activities that causes large increases in breathing or heart rate like (running or football) for at least 10 minutes continuously?	☐1. Yes<br>☐2. No<br>*(If No, go to* P13*)*	P10

11	In a typical week, on how many days do you do **vigorous-intensity** sports, fitness or recreational (leisure) activities?	Days a week	P11
12	How much time do you spend doing **vigorous-intensity** sports, fitness or recreational (leisure) activities on a typical day?	…..hrs…...mins	P12 (a-b)
13	Does you do any **moderate-intensity** sports, fitness or recreational (leisure) activities that causes small increases in breathing or heart rate such as brisk walking (cycling, swimming, volleyball) for at least 10 minutes continuously?	☐1. Yes<br>☐2. No<br>*(If No, go to* P16*)*	P13
14	In a typical week, on how many days do you do **moderate-intensity** sports, fitness or recreational (leisure) activities?	Days a week	P14
15	How much time do you spend doing **moderate-intensity** sports, fitness or recreational (leisure) activities on a typical day?	…..hrs…...mins	P15 (a-b)
**Question about sitting or reclining activities**			
The following question is about sitting or reclining at work, at home, getting to and from places, or with friends including time spent (sitting at a desk, sitting with friends, travelling in car, bus, train, reading, playing cards or watching television), but do not include time spent sleeping			
16	How much time do you usually spend sitting or reclining on a typical day?	…..hrs…...mins	P16 (a-b)

Calculation: Physical activity ………………………… Kcal (GPAC)

Interpretation: **low physical activity** ☐1. Yes ☐ 2. No

Low physical activity = male ≤383 Kcal female ≤270 Kcal

4. Slow walking speed phenotype

This test measures the walking time over a distance of 15 feets. (4.57 meters)

walk time over **4.57 meters (WS)** = …….....…. second

Interpretation: **Slow walking speed** ☐1. Yes ☐2.No

**Interpretation of results takes into account sex and height:**			
**male**	Height ≤ 173 cm.	Slow walking speed if	WS ≥ 7 seconds
	Height > 173 cm.	Slow walking speed if	WS ≥ 6 seconds
**female**	Height ≤ 159 cm.	Slow walking speed if	WS ≥ 7 seconds
	Height > 159 cm.	Slow walking speed if	WS ≥ 6 seconds

5. Decrease grip strength phenotype:

<u>**Measurement protocol**</u> Weakness was determined by grip-strength, which was measured three times on the subject's dominant side (for 3-5 seconds) with a digital hand grip dynamometer. The highest recorded value of grip-strength was taken as the maximal value.

1st time …………………… kg. 2nd time …………………. kg. 3rd time ………………….. kg.

Hand grip dynamometer (**Highest recorded value**) = **…………....... kg.**

Interpretation: **Decrease Grip strength** ☐1. Yes ☐2. No

Grip-strength dynamometer is interpreted based on sex and body mass index (BMI) taking into an account.

Sex	Interpreted as decrease grip strength when the value (kg) is
Men	
BMI $\leq$ 24 kg/m^2	**< 29**
24 < BMI $\leq$ 28 kg/m^2	**$\leq$ 30**
BMI > 28 kg/m^2	**$\leq$ 32**
Women	
BMI $\leq$ 23 kg/m^2	**< 17**
23 < BMI $\leq$ 26 kg/m^2	**$\leq$ 17.3**
26 < BMI $\leq$ 29 kg/m^2	**$\leq$ 18**
BMI > 29 kg/m^2	**$\leq$ 21**

Interpretation of frailty phenotypes: **overall frailty score (Total 5) …………….. Point**

☐ **Frail (Subject met 3 or more of the criteria)**

☐ **Pre-frail (Subject met 1 or 2 of the criteria)**

☐ **Non-frail (Subject met none of frail phenotypic criteria)**
